# Elevated soluble TNFα levels and upregulated TNFα mRNA expression in purified peripheral blood monocyte subsets associated with high-grade hepatocellular carcinoma

**DOI:** 10.1186/s12950-020-00243-7

**Published:** 2020-03-30

**Authors:** C. Martín-Sierra, R. Martins, M. Coucelo, A. M. Abrantes, R. C. Oliveira, J. G. Tralhão, M. F. Botelho, E. Furtado, M. R. Domingues, A. Paiva, P. Laranjeira

**Affiliations:** 1 Flow Cytometry Unit, Clinical Pathology Service, Centro Hospitalar e Universitário de Coimbra, Praceta Prof. Mota Pinto, Ed. S. Jerónimo, 3° piso, Coimbra, Portugal; 2 Coimbra Institute for Clinical and Biomedical Research (iCBR), Faculty of Medicine, University of Coimbra, Coimbra, Portugal; 3 Center for Innovative Biomedicine and Biotechnology (CIBB), University of Coimbra, Coimbra, Portugal; 4 Unidade Transplantação Hepática Pediátrica e de Adultos, Centro Hospitalar e Universitário de Coimbra (UTHPA, CHUC), Coimbra, Portugal; 5 Serviço de Cirurgia Geral, Unidade HBP, Centro Hospitalar e Universitário de Coimbra (CHUC), Coimbra, Portugal; 6 Instituto de Biofísica, IBILI, Faculdade de Medicina, Universidade de Coimbra, Coimbra, Portugal; 7 Coimbra Institute for Clinical and Biomedical Research (iCBR) area of Environment Genetics and Oncobiology (CIMAGO), Faculty of Medicine, University of Coimbra, Coimbra, Portugal; 8 Unidade de Hematologia Molecular, Serviço de Hematologia Clínica, Centro Hospitalar e Universitário de Coimbra (CHUC), Coimbra, Portugal; 9 Serviço de Anatomia Patológica, Centro Hospitalar e Universitário de Coimbra (CHUC), Coimbra, Portugal; 10 Mass Spectrometry Centre, Department of Chemistry & QOPNA, University of Aveiro, Campus Universitário de Santiago, Aveiro, Portugal; 11 Instituto Politécnico de Coimbra, ESTESC-Coimbra Health School, Ciências Biomédicas Laboratoriais, Coimbra, Portugal

**Keywords:** Flow cytometry, qRT-PCR, Hepatocellular carcinoma, Cholangiocarcinoma, Circulating monocytes, TNFα

## Abstract

**Background:**

Chronic inflammation is involved in the initiation and progression of various cancers, including liver cancer. The current study focuses on the characterization of the peripheral immune response in hepatocellular carcinoma (HCC) and cholangiocarcinoma (CCA) patients, before and after surgical procedure, in order to assess the effect of tumor resection in the immune system homeostasis and to determine possible prognostic factors associated with high-grade tumors. We developed a whole-blood assay to monitor immune alterations and functional competence of peripheral monocytes in a group of 10 healthy individuals (HG), in 20 HCC patients and 8 CCA patients, by multi-color flow cytometry, qRT-PCR, and ELISA techniques.

**Results:**

The qRT-PCR analysis showed an upregulation of TNFα expression by classical and intermediate monocytes purified from HCC patients presenting tumors in grade G3-G4 as compared to G1-G2 HCC patients. Moreover, ELISA assay confirmed elevated serum levels of TNFα in G3-G4 compared to G1-G2 HCC patients. A significant decrease of circulating non-classical monocytes was detected in both CCA and HCC patients before and after surgical procedure. In addition, a functional defect in circulating classical and intermediate monocytes was observed in both groups of cancer patients when compared to the HG, with partial recovery after the surgical intervention.

**Conclusions:**

This integrated analysis permitted the identification of altered functional competence of monocyte subsets in CCA and HCC patients. In addition, our results point to a potential role of TNFα as a prognostic peripheral biomarker in HCC patients, indicating the presence of high-grade tumors that should be further validated.

## Background

Liver cancer is the second most common cause of cancer-related death worldwide, with a steady increasing incidence and mortality [1] and, therefore, a major public health challenge. It is considered an immunogenic cancer because 90% of cases develop under conditions of chronic inflammation [1, 2]. This inflammation conducts to the development of tumors and it is associated with higher tumor immunogenicity. For this reason, the most suitable therapeutic strategies to be applied in these types of carcinomas would be immunotherapeutic approaches [3]. Hepatocellular carcinoma (HCC) is the most frequent liver cancer and is associated to high morbidity and mortality rates [1, 4]. It presents poor prognosis, generally due to its late presentation and thus, late diagnostic. Cholangiocarcinoma (CCA), a malignancy that originates from biliary epithelia, is an aggressive cancer with a high mortality rate [5] and, along with HCC, represents a major primary liver cancer [2]. CCA is difficult to diagnose due to its silent and nonspecific clinical features and, in most cases, the symptoms occur when the tumor has reached an advanced stage [6]. For patients with advanced disease, there are only limited therapeutic treatment options that provide limited benefits for a subset of patients. Therefore, novel therapeutic options are needed [7]. Partial surgical resection and liver transplantation are potentially curative treatments in selected patients with HCC and CCA [8, 9]. Unfortunately, a high postoperative tumor recurrence rate significantly decreases long-term survival outcomes [9].

Prognosis of cancer patients is based on tumor-related factors as well as on host-related factors, including systemic immune cell activation [10]. Given the difficulties in acquiring liver tissues, circulating biomarkers as well as comprehensive studies monitoring the peripheral immune system homeostasis, are needed. In fact, it has been demonstrated that immune monitoring of peripheral blood (PB) cells might lead to the identification of biomarkers, which could serve to predict prognosis and/or therapy response [11]. Due to its immunogenic origin, further knowledge of the changes on immune cell populations may help define the potential of the combination of therapies, such as adoptive immunotherapy and/or checkpoint inhibition [12]. Moreover, monitoring of the peripheral immune response after surgical resection will provide information to advance our understanding of the mechanisms underlying the clinical response to surgical resection.

In the present study, we have characterized, by multi-color flow cytometry, the frequency, composition and activity of circulating monocyte subsets in PB samples from HCC and CCA patients, in order to understand the immune status of these patients, and to obtain evidence about the changes in the proportion or functions of these populations, for application in the clinical diagnosis and future development of treatments to HCC and CCA.

## Results

### Alterations of peripheral blood leukocyte subsets in CCA and HCC patients

From the data obtained by flow cytometry immunophenotyping, CCA and HCC patients displayed a decrease in the frequency and absolute numbers of PB monocytes, both at T0 and T1, compared to the HG. Regarding monocyte subsets, CCA patients displayed a significant decrease in the absolute numbers of PB classical, intermediate and non-classical monocytes, both at T0 and T1, when compared to the HG, and a significant decrease in the relative frequency of non-classical monocytes (within circulating monocytes) at T0 in comparison to the HG, that was partially restored at T1. HCC patients presented a significant decrease in the relative frequency and absolute numbers of circulating non-classical monocytes, both at T0 and T1, when compared to the HG, and a significant increase in the relative frequency of classical monocytes, both at T0 and T1, as compared to the HG. Interestingly, the relative frequency of intermediate monocytes presenting high-level surface expression of HLA-DR [13] (HLADR^++^) was significantly decreased in HCC patients at T0 in comparison to the HG (Table 1).

**Table 1:** Frequency (%) and absolute numbers (cells/ μL) of peripheral blood monocyte subsets in cholangiocarcinoma (CCA) and hepatocellular carcinoma (HCC) patients, both at the time of surgical procedure (T0) and once the patients were recovered from surgery (T1), and in healthy individuals (HG). Frequencies of monocyte subsets are related to the total of monocytes

	CCA ***N*** = 8		HCC ***N*** = 20		HG ***N*** = 10
	T0	T1	T0	T1	
% Monocytes (in whole blood)	**2,4 (1,0-6,6)**^**a,b**^	**4,9 (2,2-7,0)**^**a**^	**4,2 (1,5–10)**^**a,b**^	**5,5 (2,7-9,5)**^**a**^	8,2 (5,7–12)
Monocytes/μL	**141 (51–330)**^**a**^	**219 (128–306)**^**a**^	**329 (135–850)**^**a**^	353 (127–733)	503 (285–757)
% Classical	89 (76–98)	84 (75–92)	**89 (41–97)**^**a**^	**90 (74–96)**^**a**^	83 (70–88)
Classical monocytes/μL	**139 (40–287)**^**a,b**^	**161 (111–282)**^**a**^	318 (129–1361)^c^	264 (102–601)	393 (171–651)
% Intermediate	4,4 (0,3–14)	7,3 (4,8–12)	2,5 (0,5–42)	4,8 (1,7–13)	5,5 (2,8-8,2)
Intermediate monocytes/μL	**6,1 (0,4–36)**^**a**^	**15 (8,3–17)**^**a**^	12 (0,8–194)	13 (5,8–79)	23 (17–51)
% HLADR^+^ Intermediate	32 (23–83)	44 (33–63)	**45 (16–72)**^**a**^	38 (23–80)	33 (13–56)
% HLADR^++^ Intermediate	68 (17–77)	56 (37–67)	**55 (28–84)**^**a**^	62 (20–77)	68 (44–87)
% Non-Classical	**6,0 (0,4-7,5)**^**a**^	5,0 (1,1–14)	**3,6 (1,0–22)**^**a**^	**3,5 (0,9–13)**^**a**^	11 (5,2–28)
Non-classical monocytes/μL	**6,7 (0,6–24)**^**a**^	**6,5 (1,4–24)**^**a**^	**15 (2,4–60)**^**a**^	**13 (2,6–58)**^**a**^	61 (26–84)

Independent-samples Mann-Whitney U test was performed to compare: each group of patients vs healthy group (a); CCA vs HCC (b), The Wilcoxon test was performed to compare T1 vs T0 (c), all of them with a significance level of 0.05 (*p* < 0.05). The results are given by median (minimum value-maximum value)

### Functional alterations of peripheral blood monocyte subsets in CCA and HCC patients

**Fig. 1:**
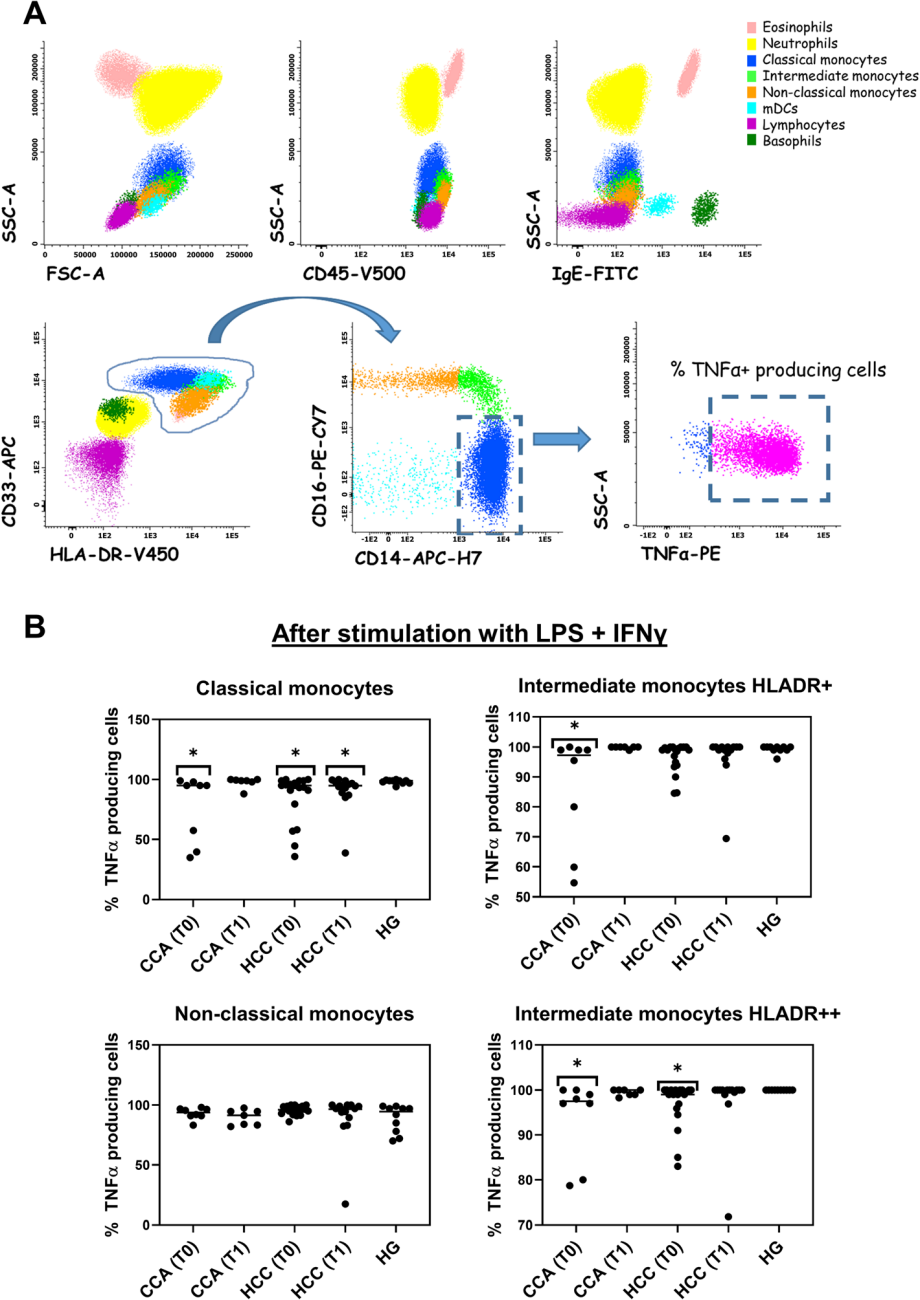
Phenotypic and functional characterization of circulating monocyte subsets. **a** Bivariate dot plot histograms illustrating the identification of circulating leukocyte subsets: eosinophils (light pink events), neutrophils (yellow events), classical monocytes (blue events), intermediate monocytes (light green events), non-classical monocytes (orange events), myeloid dendritic cells (mDCs, light blue events), lymphocytes (purple events), and basophils (green events), and an example bivariate dot plot histogram illustrating TNFα production (indicated with dashed rectangle) by classical monocytes after stimulation with LPS and IFN-γ. **b** Dot plots with the frequency of classical monocytes, intermediate monocytes with mid-level surface expression of HLA-DR (HLADR^+^), intermediate monocytes presenting high-level surface expression of HLA-DR (HLADR^++^) and non-classical monocytes producing TNFα, after stimulation with LPS plus IFNγ, in cholangiocarcinoma (CCA, *N* = 8) and hepatocellular carcinoma (HCC, *N* = 20) patients, both at T0 and T1, and in the healthy group (HG, *N* = 10). Statistically significant differences were considered when *p* < 0.05; * between the groups indicated in the figure and the HG

The different leukocyte subpopulations were identified and distinguished based on CD45 positivity, their typical forward scatter (FSC) and side scatter (SSC) light dispersion properties, and their positivity for specific membrane markers as shown in Fig. 1a. Regarding TNFα production after stimulation (Fig. 1b and Supplementary Table S2), a statistically significant decrease in the frequency of TNFα producing classical monocytes, for CCA (74% ± 29) and HCC (85% ± 20) at T0, was observed when compared to the HG (98% ± 2). HCC patients displayed a significant decrease in the frequency of TNFα producing classical monocytes at T1 (90% ± 16) as compared to the HG (98% ± 2), while no differences were found for CCA patients at T1. A statistically significant decrease in the frequency of TNFα producing intermediate monocytes with mid-level surface expression of HLA-DR (HLADR^+^) was observed for CCA patients (87% ± 20) vs. HG (99% ± 1). Additionally, both CCA (93% ± 10) and HCC (97% ± 5) patients displayed a statistically significant decrease in the frequency of TNFα producing intermediate monocytes with high-level surface expression of HLA-DR (HLADR^++^) at T0 in comparison to the HG (100% ± 0).

We did not observe significant differences when comparing patients presenting tumors classified as G1-G2 to patients presenting tumors classified as G3-G4 (data not shown).

**Fig. 2:**
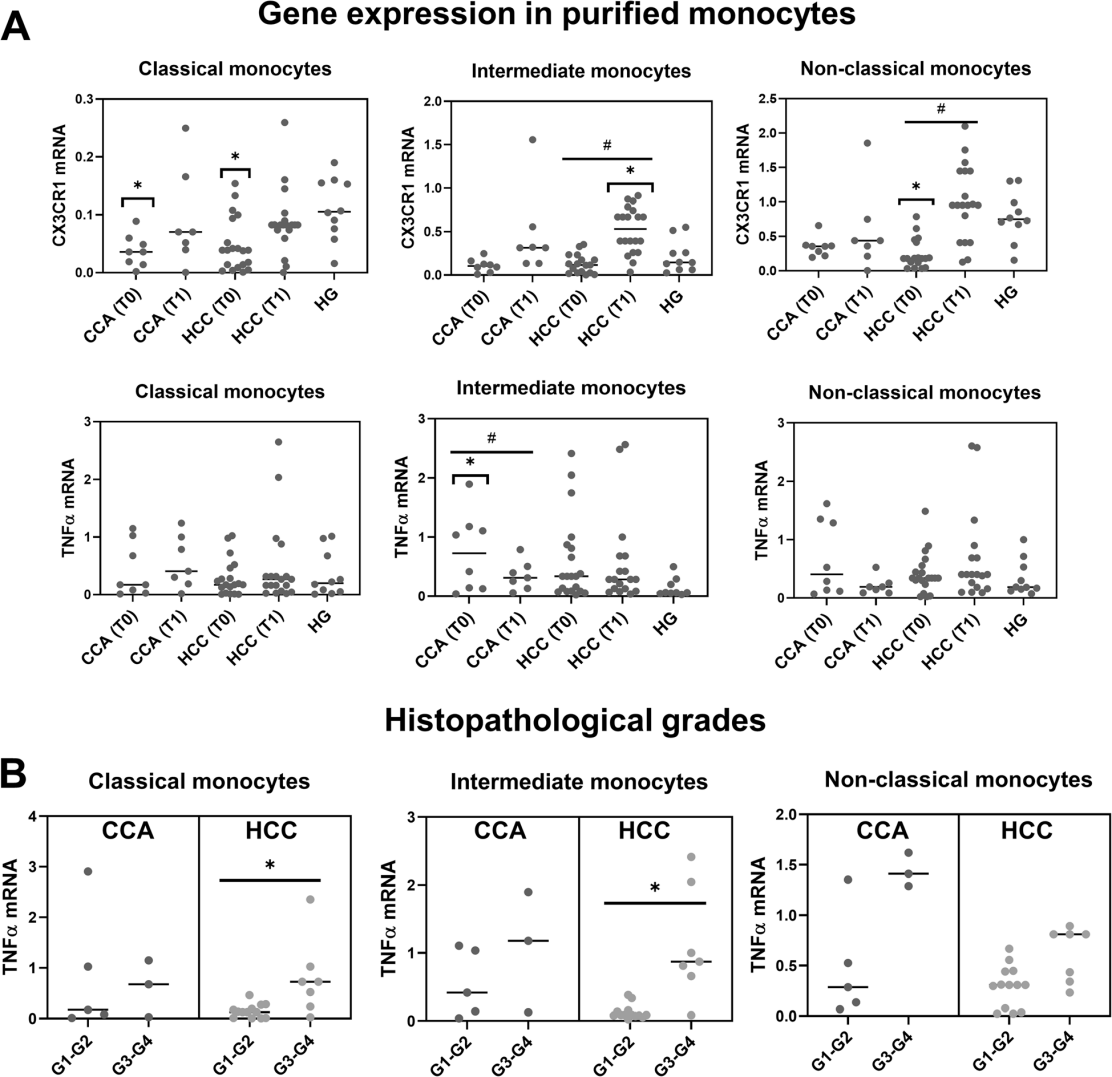
Gene expression in purified classical, intermediate and non-classical monocyte subsets. **a** Dot plots with the mRNA expression levels of CX3CR1 and TNFα in classical, intermediate and non-classical monocytes purified from CCA (N = 8) and HCC (N = 20) patients, at T0 and T1, and from the HG (*N* = 10). Statistically significant differences were considered when p < 0.05; * between the groups indicated in the figure and the HG; # between T0 and T1. **b** Dot plots with the mRNA expression levels of TNFα in classical, intermediate and non-classical monocytes purified from CCA and HCC patients, at T0, comparing G1-G2 versus G3-G4 tumor histopathological grades. Statistically significant differences were considered when p < 0.05; * between the groups indicated in the figure

After the purification of monocyte subpopulations by cell sorting, the mRNA levels of CX3CR1 and TNFα were measured by qRT-PCR. The expression levels of CX3CR1 mRNA among classical monocytes were significantly decreased in both groups of cancer patients at T0 when compared to the HG, with partial recovery at T1 in both groups of cancer patients (Fig. 2a). A significant increase in the expression levels of CX3CR1 mRNA among intermediate monocytes was detected in HCC patients at T1. Moreover, the expression levels of CX3CR1 mRNA by non-classical monocytes were significantly decreased in HCC patients at T0 when compared to the HG, with a recovery at T1. The same pattern was observed in CCA patients but without reaching statistical significance.

On the other hand, no differences were observed on TNFα mRNA expression among classical and non-classical monocytes, in CCA or HCC patients, when compared to the HG. Nevertheless, CCA patients displayed a significant increase of TNFα mRNA in intermediate monocytes at T0 in comparison to the HG, while at T1, TNFα mRNA levels approach to those observed in HG (Fig. 2a).

Taking into account the histopathological grade of the tumors, HCC patients presenting tumors classified as grade G3-G4 displayed significantly higher levels of TNFα mRNA in classical and intermediate monocytes at T0 in comparison to HCC patients with G1-G2 tumors; non-classical monocytes displayed the same tendency, without reaching statistical significance (Fig. 2b). The same pattern was observed in CCA patients but without reaching statistical significance (Fig. 2b).

### Analysis of chemokines and cytokines serum levels in CCA and HCC patients

**Fig. 3:**
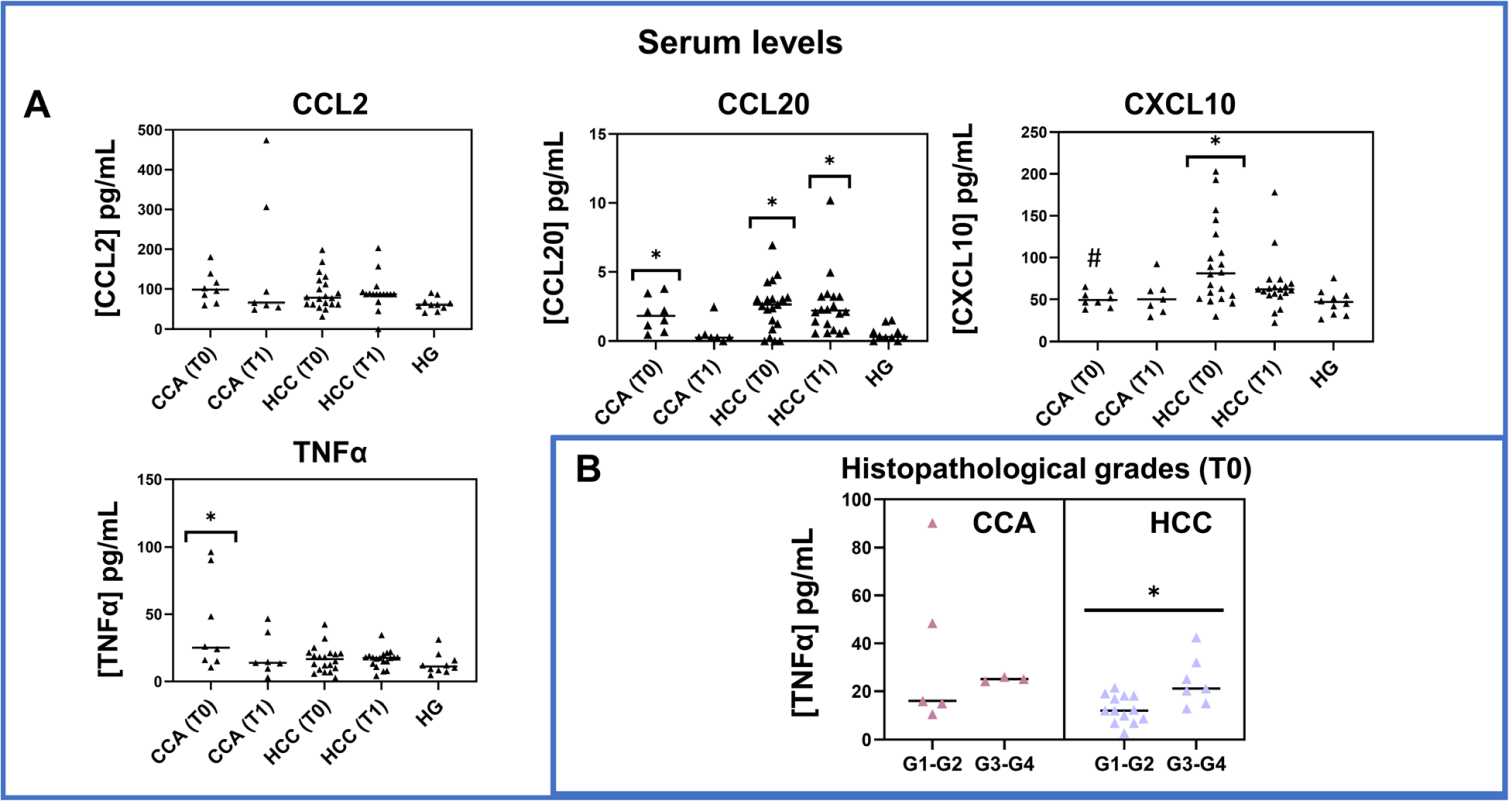
Quantification of the serum levels of CCL2, CCL20, CXCL10 and TNFα. **a** Dot plots with serum levels of CCL2, CCL20, CXCL10 and TNFα, measured by ELISA in serum samples from cholangiocarcinoma (CCA, N = 8) and hepatocellular carcinoma (HCC, N = 20) patients, at T0 and T1, and from the healthy group (HG, N = 10). Statistically significant differences were considered when p < 0.05; *between the groups indicated in the figure and the HG; #between CCA and HCC at the same time point. **b** Dot plots with serum levels of TNFα, measured in CCA (N = 8) and HCC (N = 20) patients, at T0, comparing G1-G2 versus G3-G4 tumor histopathological grades. Statistically significant differences were considered when p < 0.05; * between the groups indicated in the figure

With regard to the chemokines analyzed by ELISA, elevated serum levels of CCL20 were found in both CCA and HCC patients at T0, compared to the HG. While CCA patients showed a partial recovery at T1, HCC patients’ CCL20 levels remained high at T1 (Fig. 3a). Circulating levels of CXCL10 were also increased in HCC patients, at T0, in comparison to the HG, followed by a partial recovery at T1. Conversely, no significant differences were observed for CCL2 among the studied groups (Fig. 3a).

Concerning serum levels of TNFα, CCA patients displayed a significant increase of TNFα at T0, in comparison to the HG, partially restored at T1, while no significant differences were observed for HCC patients (Fig. 3a).

Notably, serum levels of TNFα were significantly higher in G3-G4 compared to G1-G2 HCC patients (at T0). A similar pattern was observed in CCA patients without reaching statistical significance (Fig. 3b).

## Discussion

The current study has identified a functional defect on some circulating monocyte subsets in HCC and CCA patients, regarding their capability to produce TNFα under stimulation, displaying a partial recovery after surgical procedure. Moreover, a peripheral pro-inflammatory state in HCC and CCA patients was observed, and, a potential role for TNFα circulating levels, as well as TNFα mRNA expression by classical and intermediate monocytes, as prognostic peripheral biomarkers in HCC patients, indicating the presence of high-grade (G3 or G4) tumors, was revealed.

Inflammatory factors affect nearly all the stages of tumor development and the effectiveness of the applied therapies. TNFα is considered one of the most important inflammatory mediators of the cancer-associated inflammatory networks. In this regard, preclinical studies in breast cancer have suggested that TNFα promotes tumor growth in vivo and could be considered a therapeutic target [14]. In the present study, we pointed out an upregulation of TNFα mRNA expression in peripheral classical, intermediate and non-classical monocytes purified from HCC patients presenting tumors classified as grade G3-G4, as compared to G1-G2 HCC patients. Moreover, in line with these results, we detected a significant increase of TNFα serum levels in HCC patients with tumors in grade G3-G4 as compared to grade G1-G2 HCC. In this regard, further studies will be needed to validate the potential use of TNFα serum levels as a prognostic factor or indicator of histopathological grading. Supporting this idea, it has been reported that aberrant elevated TNFα levels might promote not only tumor growth, but also invasion and poor prognosis [15]. Additionally, a previous work in a rat liver cancer model demonstrated that TNFα inhibition and deletion could decrease tumor incidence and showed that clinically TNFα expression was correlated to hepatic progenitor cells activation and HCC recurrences [16].

Regarding the characterization of circulating monocytes, we observed a significant decrease in the frequency and absolute counts of PB monocytes, both in CCA and HCC patients, at T0, as compared to the HG. Interestingly, values continued to be diminished comparatively to the HG after surgical procedure (at T1). The observed decrease in the frequency of circulating monocytes could be associated to an increase in the frequencies of circulating neutrophils since previous works have identified elevated levels of circulating neutrophils that were significantly and independently associated with poorer prognosis in HCC [17–19]. Peripheral blood monocytes constantly enter the liver to replenish hepatic macrophages and dendritic cells, playing important roles in the pathogenesis of inflammatory disorders [20]. Our previous findings had already shown altered frequencies and altered absolute numbers of circulating FcεRI^+^ monocytes and myeloid dendritic cells in CCA and HCC patients, as well as functional defects in both cell subsets [21]. Thus, we wondered whether other monocyte subsets might be affected in these patients. The results of the present study extend our previous findings, showing alterations on monocytes function, as well as a significant decrease in the relative frequency and absolute numbers of circulating non-classical monocytes both in CCA and HCC patients. These values remained diminished as compared to the HG at T1, excepting the frequency of non-classical monocytes in CCA patients, that was partially restored after tumor resection. Non-classical monocytes “patrol” the vasculature via a mechanism that requires the fractalkine receptor CX3CR1, covering apoptotic endothelial cells and sensing danger signals coming from the tissue [22]. A previous work has shown that this population of monocytes reduces tumor metastasis by recruiting NK cells [23]. Therefore, we hypothesized that the observed decrease in this monocyte subset may be having a role in cancer progression. Additionally, it has been suggested that non-classical monocytes are crucial regulators in the pathogenesis of chronic liver disease in humans, by the secretion of abundant cytokines, perpetuating chronic inflammatory processes within the liver and by directly activating hepatic stellate cells that, in turn, can secrete multiple chemokines for monocyte recruitment into the liver [24]. Therefore, indicating that the modulation of monocyte-subset recruitment into the liver may represent possible novel approaches for interventions targeting pro-inflammatory actions of monocyte subsets in liver cancer.

When evaluating monocyte function, we observed, at T0, a significant decline in TNFα production by classical and HLADR^++^ intermediate monocyte subsets, under LPS plus IFNγ stimulation, in both CCA and HCC patients as compared to the HG, whereas no significant differences were detected for non-classical monocytes. TNFα production by HLADR^+^ intermediate monocyte was also affected in CCA patients before tumor resection, but not in HCC patients. The frequencies of TNFα-producing cells seem to be restored after tumor resection, excepting classical monocyte subsets that displayed a significant decrease in TNFα production in HCC patients at T1. These data indicate a functional defect in classical and intermediate monocytes in both, CCA and HCC patients that it is partially restored for intermediate monocytes at T1. In line with these results, previous studies in other carcinomas have reported a diminished percentage of TNFα-producing CD14^+^ cells in PB of lung adenocarcinoma patients, showing that malignant cells inhibited the capability of monocytes to produce TNFα [25]. Thus, the decreased TNFα expression, observed in classical and intermediate monocytes, may be caused by tumor cells, but also, may influence tumor progression and relapse, since an altered response of innate immune cells might underscore a reduced capacity to mount an efficient antitumor immune response [26]. Monocytes can differentiate into a variety of macrophage or dendritic cell subtypes that can either activate or inhibit the immune response [27], and it has been shown that CX3CR1 is rapidly downregulated during monocytes differentiation [28]. In relation with this process, classical and non-classical monocytes purified from CCA and HCC patients presented a downregulation of the mRNA expression of the fractalkine receptor, CX3CR1, at T0, when compared to the HG, with partial recovery at T1, indicating a possible differentiation process in these monocyte subsets occurring before surgical procedure. Additionally, CX3CR1 expression could participate in promoting cell-to-cell interactions with an inflamed endothelium, as well as increasing monocytes survival [28]. We also detected, in intermediate monocytes from CCA patients, an upregulation of TNFα mRNA expression at T0 that was restored after surgical procedure, which is further supported by the results obtained from the measurement of serum TNFα levels, in which we detected significantly elevated TNFα serum levels in CCA patients at T0, that were restored at T1. Indicating an important role of peripheral TNFα in CCA associated inflammatory response.

In addition, we detected elevated circulating levels of CXCL10 in HCC patients, before surgery, that were only partially restored at T1. CXCR3 is the receptor for CXCL10, a member of the CXC chemokine family with pro-inflammatory and anti-angiogenic properties [29] that has been associated with a variety of human diseases, including tumor development, metastasis, and dissemination. More importantly, CXCL10 has been identified as a major biological marker mediating disease severity and may be used as a prognostic indicator for various diseases [30], thus, its possible role as disease severity biomarker for liver cancers should be further evaluated. Moreover, circulating levels of CCL20 in HCC patients before and after tumor resection were significantly increased, in comparison to the HG, as well as in CCA patients at T0, which presented a partial recovery after tumor resection. CCL20, alternatively called liver activation regulated chemokine (LARC), is a strong chemoattractant for leukocytes expressing its sole receptor CCR6 [31]. Many studies have shown that the CCL20/CCR6 axis contribute to the initiation and progression of HCC by mediating the migration of circulating T cells into the tumor microenvironment [32]. Thus, the observed increase in the circulating levels of CCL20 indicates a probable migration of T cells to the tumor microenvironment of these carcinomas.

The detected increase in the circulating levels of CCL20, CXCL10, and TNFα suggests a peripheral pro-inflammatory state in HCC and CCA patients and an activate state for monocyte subsets although classical and intermediate monocyte subsets displayed a functional defect at T0 and, therefore, a limited capacity to respond under further stimulation processes.

## Conclusions

This integrated analysis permitted the identification of different immune background in CCA and HCC patients, as well as altered functional competence of circulating monocytes in these carcinomas. Intriguingly, most of these alterations appear to be restored in CCA patients after tumor resection, however, most of them remained in HCC patients after surgical procedure. Moreover, this work has demonstrated that flow cytometry serves as a powerful analytical platform for the rapid characterization of individual cells within heterogenic cell populations and it may help in monitoring functional competence of immune cell populations to better evaluate immune dysfunctions in cancer patients. This approach could be applied in clinical routine to evaluate innate and adaptive immunity and to monitor responses to different treatments. In addition, the identified immune alterations should be further studied to consider clinically relevant therapeutic targets. A better understanding of the potential of inflammation and innate immunity to inhibit tumor progression should lead to the development of new and improved immunomodulatory approaches for the treatment of liver carcinomas. Notably, our results point out a potential role for TNFα as a prognostic factor in HCC patients indicating the presence of high-grade tumors. However, further studies should be conducted with larger sample sizes to validate the findings we have presented and correlate these findings with post-surgery outcomes after long-term follow-up.

## Methods

The aim of the present study was to characterize, by multi-color flow cytometry, the frequency, composition and activity of circulating monocyte subsets in PB samples from HCC and CCA patients, in order to understand the immune status of these patients, and to obtain evidence about the changes in the proportion or functions of these populations, for application in the clinical diagnosis and future development of treatments to HCC and CCA.

### Patients and healthy individuals

PB samples were collected from 20 patients with HCC (3 women and 17 men; average age: 62.2 ± 14.5 years) and 8 patients with CCA (5 women and 3 men; average age: 61.0 ± 14.7 years), just before the beginning of the surgical resection (T0), and once the patient was completely recovered from the surgical procedure (T1). In addition, a group of 10 age- and sex-matched healthy individuals was included in the present study as a control group (HG). None of the patients included in this study took any medication before surgical procedure nor at T1. Nonetheless, 7 HCC patients took tacrolimus following liver transplantation which targets T lymphocytes [33, 34] but has no effect over monocyte function [35].

Patients were classified according to the 8th TNM classification and their clinical background is summarized in Table 2.

**Table 2:** Clinical data from hepatocellular carcinoma (HCC) and cholangiocarcinoma (CCA) patients enrolled in this study. Number of patients and frequencies (%) are indicated

		CCA ***N*** = 8	HCC ***N*** = 20
**TNM**	Stage I	1 (13%)	3 (15%)
	Stage II	4 (50%)	15 (75%)
	Stage IIIA	0 (0%)	1 (5%)
	Stage IV	3 (38%)	1 (5%)
**Histologic grade (G)**	G1	2 (25%)	2 (10%)
	G2	3 (38%)	11 (55%)
	G3	3 (38%)	6 (30%)
	G4	0 (0%)	1 (5%)
**HBsAg**	Positive	0 (0%)	1 (5%)
**HCV**	Positive	0 (0%)	6 (30%)
**Vascular microinvasion**	Positive	–	8 (40%)
**Cirrhosis**		0 (0%)	15 (75%)
**Relapse**		3 (38%)	1 (5%)
**Death**		3 (38%)	2 (10%)
**Liver Transplant**		0 (0%)	7 (35%)

We did not identify any significant difference between HCC patients presenting liver cirrhosis and HCC patients without liver cirrhosis for all the parameters analyzed in this study. Therefore, all HCC patients were included in the same group.

### In vitro stimulation of cytokine production by circulating monocytes

To evaluate the TNFα production by classical, intermediate and non-classical monocytes, PB samples were collected from participants and healthy individuals into lithium heparin (Becton Dickinson Biosciences, BD, San Jose, CA, USA) and stimulated with lipopolysaccharide (LPS) plus interferon gamma (IFNγ), as previously described in Martín-Sierra et al. 2019 [21]. Brefeldin-A was added to inhibit protein transport in Golgi complex, as it redistributes intracellularly produced proteins from the cis/medial Golgi complex to the endoplasmic reticulum. All samples were then incubated in a 5% CO_2_ humid atmosphere, at 37 °C, for 6 h.

Immunophenotypic analysis was performed by using a seven-color monoclonal antibodies (mAbs) combination, detailed in Supplementary Table S1 (tube 1). Samples were aliquoted (300 μL) and stained with the mAbs for surface proteins antigens (CD45, HLA-DR, IgE, CD16, CD33 and CD14). After extracellular staining, we followed the protocol previously described in Martín-Sierra et al. 2019 [21].

### Flow cytometry data acquisition and analysis

The stained samples were acquired in a FACSCanto II flow cytometer (BD) and the data were analyzed with Infinicyt 1.8 software (Cytognos SL, Salamanca, Spain).

For the identification of the distinct monocyte subsets, we used the following gating strategy: we selected the monocyte population by its characteristic FSC/SSC light dispersion properties together with high expression of CD45, HLA-DR, and CD33. Within monocytes, classical, intermediate and non-classical monocyte subpopulations were distinguished based mainly on the expression levels of CD16 and CD14. Classical monocytes express high levels of CD14 in the absence of CD16, together with high expression of CD33 and HLA-DR; intermediate monocytes express high levels of CD14 as well, but display an increasing expression of CD16, associated to a slight decrease of CD33 expression, compared to classical monocytes; in turn, non-classical monocytes show CD16 positivity with a decreasing expression of CD14, they present the highest expression of CD45 along with the lowest expression of CD33 among the three monocyte subpopulations (as displayed in Fig. 1a).

When these cell subsets were characterized and identified in the Infinicyt software, we plotted each population independently to determine TNFα production levels by each subset of monocytes (as indicated in the last plot of Fig. 1a).

### Cell purification by fluorescence-activated cell sorting

Classical (CD14^++^CD16^−^), intermediate (CD14^++^CD16^+^) and non-classical (CD14^+^CD16^++^) monocyte subpopulations from PB were purified by fluorescence-activated cell sorting (FACS), using a FACSAria III flow cytometer (BD) according to their typical phenotype. Thus, the four-color mAbs combination used (Supplementary Table S1, tube 2) allowed the identification of each monocyte subset.

For subsequent mRNA expression analysis, purified cell populations were prepared and stored as previously described in our laboratory [36].

### RNA isolation and quantitative real-time reverse transcriptase-polymerase chain reaction (qRT-PCR)

We used the RNeasy™Micro Kit (Qiagen) according to the manufacturer’s instructions to extract and purify total RNA content from each cell subpopulation. Reverse transcription and relative quantification of gene expression was performed as previously described in our laboratory [36], using optimized primers for TNFα, CX3CR1 and endogenous control glyceraldehyde 3-phosphate dehydrogenase (GAPDH) (Qiagen), according to the manufacturer’s instructions.

### Assessment of cytokine and chemokine serum concentrations

PB samples were collected from patients and healthy individuals into VACUETTE Serum Gel tubes (Greiner-Bio-One, Kremsmünster, Austria) and were centrifuged for 10 min at 2000 g. Subsequently, serum samples were subdivided into small aliquots to be stored at − 80 °C until tested for cytokine and chemokines levels. ELISA kits were used to determine monocyte chemotactic protein-1 (MCP-1 or CCL2, Thermo Scientific), MIP-3a (CCL20, Thermo Scientific), IP-10 (CXCL10, Thermo Scientific) and TNFα (Thermo Scientific) serum levels, according to the manufacturer’s instructions*.*

### Statistical analysis

Data are presented as the mean values with their standard deviation or as the median with the minimum and maximum values. The non-parametric Mann-Whitney and Kruskal-Wallis multiple comparison tests were employed, using the Statistical Package for Social Sciences software (SPSS, version 25, IBM, Armonk, NY, USA). Moreover, the non-parametric Wilcoxon signed-rank test of non-independent data was performed to compare T1 vs T0. Statistical significance was considered when *p* < 0.05. GraphPad Prism software was used to create the graphics.

## Supplementary information

**Additional file 1: Table S1.** Panel of mAb reagents (with clones and commercial source) used for the immunophenotypic characterization and for cell purification by fluorescence-activated cell sorting of tumor cells and immune cells. **Table S2.** Absolute numbers (cells/ μL) and frequencies (%) of TNFα producing peripheral blood monocyte subsets in cholangiocarcinoma (CCA) and hepatocellular carcinoma (HCC) patients, both at the time of surgical procedure (T0) and once the patients were recovered from surgery (T1), and in healthy individuals (HG).

## Data Availability

The authors declare that the main data supporting the results of the present study are available within the article and its Supplementary Information files. Extra data are available from the corresponding author upon request.
