# Supplementary material for: Elevated soluble TNFα levels and upregulated TNFα mRNA expression in purified peripheral blood monocyte subsets associated with high-grade hepatocellular carcinoma
Source: J Inflamm (Lond). 2020 Mar 30;17:14. doi: 10.1186/s12950-020-00243-7 (PMC7106708; doi:10.1186/s12950-020-00243-7)
Supplement: Supplementary file 1 — Additional file 1: Table S1. Panel of mAb reagents (with clones and commercial source) used for the immunophenotypic characterization and for cell purification by fluorescence-activated cell sorting of tumor cells and immune cells. Table S2. Absolute numbers (cells/ μL) and frequencies (%) of TNFα producing peripheral blood monocyte subsets in cholangiocarcinoma (CCA) and hepatocellular carcinoma (HCC) patients, both at the time of surgical procedure (T0) and once the patients were recovered from surgery (T1), and in healthy individuals (HG). [file 12950_2020_243_MOESM1_ESM.docx]

**Elevated soluble TNFα levels and upregulated TNFα mRNA expression in purified peripheral blood monocyte subsets associated with high-grade hepatocellular carcinoma**

Martín-Sierra C.^1,2,3,*^, Martins R.^4,5,6,7,*^, Coucelo M.^8^, Abrantes A.M.^6,7^, Oliveira R.C.^6,7,9^, Tralhão J.G.^4,5,6,7^, Botelho M.F.^6,7^, Furtado E.^4^, Domingues MR.^10^, Paiva A.^1,2,3,11,#^, Laranjeira P.^1,2,3^

1 - Unidade de Gestão Operacional em Citometria, Centro Hospitalar e Universitário de Coimbra (CHUC, Portugal).

2 - Coimbra Institute for Clinical and Biomedical Research (iCBR), Faculty of Medicine, University of Coimbra (Coimbra, Portugal).

3 - Center for Innovative Biomedicine and Biotechnology (CIBB), University of Coimbra, Portugal.

4 - Unidade Transplantação Hepática Pediátrica e de Adultos, Centro Hospitalar e Universitário de Coimbra (UTHPA, CHUC, Portugal).

5 - Serviço de Cirurgia Geral, Unidade HBP, Centro Hospitalar e Universitário de Coimbra (CHUC, Portugal).

6 - Instituto de Biofísica, IBILI, Faculdade de Medicina, Universidade de Coimbra (Coimbra, Portugal).

7 - Coimbra Institute for Clinical and Biomedical Research (iCBR) area of Environment Genetics and Oncobiology (CIMAGO), Faculty of Medicine, University of Coimbra, 3000-548 (Coimbra, Portugal).

8 - Unidade de Hematologia Molecular, Serviço de Hematologia Clínica, Centro Hospitalar e Universitário de Coimbra (CHUC, Portugal).

9 - Serviço de Anatomia Patológica, Centro Hospitalar e Universitário de Coimbra (CHUC, Portugal).

10 - Mass Spectrometry Centre, Department of Chemistry & QOPNA, University of Aveiro, Campus Universitário de Santiago (Aveiro, Portugal).

11 - Instituto Politécnico de Coimbra, ESTESC-Coimbra Health School, Ciências Biomédicas Laboratoriais (Portugal).

*Shared position

**# Corresponding author:**

Artur Paiva

Flow Cytometry Unit, Clinical Pathology Service, Centro Hospitalar e Universitário de Coimbra, Praceta Prof. Mota Pinto, Ed. S. Jerónimo, 3° piso, 3001-301, Coimbra, Portugal.

Telephone: +351 239400563

Fax: +351 239824416

E-mail: [artur.paiva@chuc.min-saude.pt](mailto:artur.paiva@chuc.min-saude.pt)

ORCID: 0000-0002-6562-5859

**Supplementary material**

**Table S1.** Panel of mAb reagents (with clones and commercial source) used for the immunophenotypic characterization and for cell purification by fluorescence-activated cell sorting of tumor cells and immune cells.

| **Tube** | **V450** | **V500** | **FITC** | **PE** | **PE-Cy7** | **APC** | **APC-H7** |
| --- | --- | --- | --- | --- | --- | --- | --- |
| **1** | **HLA-DR**  L243  BD | **CD45**  2D1  BD | **IgE**  BE5  EXBIO Praha | **TNFα**  MAb11  BD | **CD16**  3G8  BD | **CD33**  P67.6  BD | **CD14**  MφP9  BD |
| **2** | **HLA-DR**  L243  BD | **CD45**  2D1  BD |  |  | **CD16**  3G8  BD |  | **CD14**  MφP9  BD |

Commercial sources: BD (Becton Dickinson Biosciences, San Jose, CA, USA), EXBIO Praha (Vestec, Czech Republic). APC, allophycocyanin; APC-H7, allophycocyanin-hilite 7; FITC, fluorescein isothiocyanate; PE, phycoerythrin; PE-Cy7, phycoerythrin-cyanine 7.

**Table S2.** Absolute numbers (cells/ µL) and frequencies (%) of TNFα producing peripheral blood monocyte subsets in cholangiocarcinoma (CCA) and hepatocellular carcinoma (HCC) patients, both at the time of surgical procedure (T0) and once the patients were recovered from surgery (T1), and in healthy individuals (HG).

|  | **CCA**  **N = 8** | | **HCC**  **N = 20** | | **HG**  **N = 10** |
| --- | --- | --- | --- | --- | --- |
|  | **T0** | **T1** | **T0** | **T1** |  |
|  |  |  |  |  |  |
| % TNFα producing classical monocytes | **74% ± 29^a^** | 97% ± 5 | **85% ± 20^a^** | **90% ± 16^a^** | 98% ± 2 |
| [TNFα producing classical monocytes]/μL | **87 ± 76^a^** | **171 ± 51^a^** | 244 ± 285 | 284 ± 150 | 386 ± 120 |
| % TNFα producing HLADR^+^ Intermediate monocytes | **87% ± 20^a^** | 100% ± 1 | 96% ± 5 | 97% ± 8 | 99% ± 1 |
| [TNFα producing HLADR^+^ Intermediate monocytes]/μL | 1.7 ± 5.7 | 6.4 ± 1.7 | 4.9 ± 17 | 8.2 ± 12 | 7.2 ± 1.9 |
| % TNFα producing HLADR^++^ Intermediate monocytes | **93% ± 10^a^** | 100% ± 1 | **97% ± 5^a^** | 98% ± 7 | 100% ± 0 |
| [TNFα producing HLADR^++^ Intermediate monocytes]/μL | 3.7 ± 7.2 | 8.2 ± 2.1 | 5.8 ± 29 | 8.3 ± 15 | 17 ± 10 |
| % TNFα producing non-classical monocytes | 93% ± 5 | 89% ± 7 | 96% ± 4 | 89% ± 22 | 89% ± 11 |
| [TNFα producing non-Classical]/μL | **5.5 ± 8.0 ^a,b^** | **4.8 ± 4.7^a,b^** | **13 ± 18 ^a,b^** | **11 ± 17^a,b^** | 47 ± 21 |
|  |  |  |  |  |  |

Independent-samples Mann-Whitney U test was performed to compare: each group of patients vs healthy group (a); CCA vs HCC (b), The Wilcoxon test was performed to compare T1 vs T0 (c), all of them with a significance level of 0.05 (p<0.05).
